# Interlimb Reflexes Induced by Electrical Stimulation of Cutaneous Nerves after Spinal Cord Injury

**DOI:** 10.1371/journal.pone.0153063

**Published:** 2016-04-06

**Authors:** Jane E. Butler, Sharlene Godfrey, Christine K. Thomas

**Affiliations:** 1 The Miami Project to Cure Paralysis, University of Miami Miller School of Medicine, Miami, Florida, United States of America; 2 Department of Neurological Surgery, University of Miami Miller School of Medicine, Miami, Florida, United States of America; 3 Department of Physiology and Biophysics, University of Miami Miller School of Medicine, Miami, Florida, United States of America; 4 Neuroscience Research Australia, Sydney, Australia; 5 University of New South Wales, Sydney, Australia; University of Szeged, HUNGARY

## Abstract

Whether interlimb reflexes emerge only after a severe insult to the human spinal cord is controversial. Here the aim was to examine interlimb reflexes at rest in participants with chronic (>1 year) spinal cord injury (SCI, n = 17) and able-bodied control participants (n = 5). Cutaneous reflexes were evoked by delivering up to 30 trains of stimuli to either the superficial peroneal nerve on the dorsum of the foot or the radial nerve at the wrist (5 pulses, 300 Hz, approximately every 30 s). Participants were instructed to relax the test muscles prior to the delivery of the stimuli. Electromyographic activity was recorded bilaterally in proximal and distal arm and leg muscles. Superficial peroneal nerve stimulation evoked interlimb reflexes in ipsilateral and contralateral arm and contralateral leg muscles of SCI and control participants. Radial nerve stimulation evoked interlimb reflexes in the ipsilateral leg and contralateral arm muscles of control and SCI participants but only contralateral leg muscles of control participants. Interlimb reflexes evoked by superficial peroneal nerve stimulation were longer in latency and duration, and larger in magnitude in SCI participants. Interlimb reflex properties were similar for both SCI and control groups for radial nerve stimulation. Ascending interlimb reflexes tended to occur with a higher incidence in participants with SCI, while descending interlimb reflexes occurred with a higher incidence in able-bodied participants. However, the overall incidence of interlimb reflexes in SCI and neurologically intact participants was similar which suggests that the neural circuitry underlying these reflexes does not necessarily develop after central nervous system injury.

## Introduction

After spinal cord injury (SCI), trivial cutaneous stimuli on the leg are an easy way to trigger local spasms which can spread to many muscles including the arms [1]. These types of interlimb connections have been studied after acute and chronic SCI [2–6] and are thought to result from the strengthening of existing connections (for review see [7]), and/or regenerative sprouting after injury that may result in the formation of new neural connections [2–5]. The idea of new growth after injury is supported by the argument that these reflexes appear after a relatively long time in humans, the responses increase in intensity over time [5] and data from animal studies showing changes in intra spinal circuitry after SCI [8–13], (for review see [14]). Alternatively, pre-existing ascending and descending propriospinal connections may become more prominent after SCI [6] where, in the absence of descending corticospinal connections, the overall level of spinal cord excitability appears to increase compared to that in able-bodied controls [15–18] after the initial period of spinal shock [19]. This may be a result of increased spinal excitation and/or decreased spinal inhibition [15,18,20–22], or changes in the intrinsic properties of the cells [13].

Interlimb reflexes have been demonstrated in able-bodied participants using electrical stimuli of cutaneous afferents [23–26], mixed nerves [27–32] and passive joint movement [33]. Stimuli applied to nerves in one lower limb can cause contralateral interlimb reflex responses in the other lower limb and ascending interlimb reflexes in the upper limbs bilaterally. Stimuli applied to an upper limb or the face can cause descending interlimb reflex responses in the lower limbs. However, to demonstrate these interlimb reflexes, these studies have required that the excitability of the spinal cord is raised either by having the participants voluntarily contract the test muscles (e.g. [24,25,27,28,30,32]) or by testing with a concurrent excitatory reflex (e.g. [27,28,31]). Interlimb reflexes have never been demonstrated previously in able-bodied participants with the muscles at rest.

For individuals with acute and chronic SCI, ascending interlimb reflexes (lower to upper limbs) have been repeatedly shown in resting muscles in response to natural cutaneous stimuli and electrical stimulation of peripheral nerves [1–6]. These reflexes are thought to be due to sprouting of disrupted ascending sensory fibres from lower limbs that connect to upper limb motoneurons caudal to the spinal lesion. This may occur directly or via interneurons and/or propriospinal neurons. In contrast, descending interlimb reflexes (upper to lower limbs) have not been demonstrated after SCI. When many muscles are paralyzed after SCI it is difficult to raise the excitability of the spinal cord in a controlled way. Therefore, our aim was to quantify and compare the interlimb reflexes evoked in resting muscles by upper and lower limb cutaneous nerve stimulation in participants with chronic cervical SCI and in uninjured able-bodied control participants. By testing interlimb reflexes during muscle relaxation we matched the level of muscle activity for each group. We have also used a stimulation protocol known to evoke interlimb reflexes in active muscles in able-bodied participants [25].

## Materials and Methods

### Participants

Seventeen participants with cervical SCI were studied, 14 men and 3 women (21–46 years, mean ± SEM: 34 ± 2 years). All had chronic spinal cord injury (1–26 years post injury, mean ± SEM: 11 ± 1 years) at C4 (n = 7), C5 (n = 4) or C6 (n = 6), as defined by American Spinal Injury Association criteria [34]. Additional information on the injury classification, cause and medication taken by the participants with SCI is given in Table 1. Five able-bodied control participants, four men and one woman (26–46 years, mean ± SEM: 34 ± 5 years), with no known neuromuscular impairment were studied with the same protocol. The five able-bodied control participants were included to demonstrate the possibility that excitatory interlimb and within-limb reflexes could be evoked in muscles at rest with the stimulation but this number is too small to give a good estimate of the proportion of able-bodied participants in which they can be evoked.

**Table 1:** Participant Description.

Group	Sex	Age	Cause	SCI Level	AIS	Duration	Medication
		Years			Classification	Years	
SCI	M	32	GSW	C4	A	12	----------
SCI	M	32	MVA	C4	A	11	Diazepam
SCI	F	46	Sports	C4	A	6	Baclofen
SCI	M	42	MVA	C4	A	7	----------
SCI	M	23	Fall	C4	A	6	Diazepam
SCI	M	32	Diving	C4	A	10	Baclofen, Diazepam
SCI	M	32	Diving	C4	A	7	Baclofen
SCI	M	37	Diving	C5	A	10	Baclofen, Diazepam
SCI	M	39	Diving	C5	A	16	Baclofen
SCI	M	34	MVA	C5	A	10	Baclofen, Diazepam
SCI	M	30	Fall	C5	A	9	Baclofen
SCI	M	40	Fall	C6	A	12	Baclofen
SCI	F	46	Bicycle	C6	A	26	----------
SCI	M	22	Bicycle	C6	B	9	----------
SCI	M	33	MVA	C6	A	12	Baclofen
SCI	M	21	MVA	C6	A	1	Baclofen
SCI	F	37	MVA	C6	A	16	----------
Mean ± SEM		34 ± 2				11 ± 1	
Control	M	26					----------
Control	M	45					----------
Control	F	27					----------
Control	M	46					----------
Control	M	28					----------
Mean ± SEM		34 ± 5					

Table 1: SCI: Spinal cord injury; M: male; F: female; GSW: gunshot wound; MVA: motor vehicle accident; C: cervical spinal level; AIS: American Spinal Cord Injury Association Impairment Scale A (motor and sensory complete) or B (motor complete, sensory incomplete)

All participants gave informed written consent to participate in the experiments, which were approved by the University of Miami Institutional Review Board and conducted in accordance with the Declaration of Helsinki.

### Experimental setup

Each participant with a SCI remained in his or her wheelchair for the duration of the experiment. Each able-bodied control participant sat relaxed in a chair. Electromyographic activity (EMG) was recorded with pairs of disposable surface electrodes (Cleartrace, Medtronic Andover Medical, Inc., Haverhill, MA) placed approximately 4 cm apart on the mid-belly of eight different muscles bilaterally (total of 16 recording sites). The 4 muscles examined in each arm were biceps brachii, triceps brachii, wrist flexors (FCR), and wrist extensors (ECR). The 4 muscles examined in each leg were hamstrings (biceps femoris), quadriceps femoris (vastus lateralis), tibialis anterior and soleus. All of the EMG signals were amplified (Grass P511, AstroMed Inc., West Warwrick, RI), filtered (30–1000 Hz) and sampled online at 3000 Hz using a SC/Zoom acquisition and analysis system (Umeå University, Sweden). Data were sampled for 80 ms prior to each train of pulses and for 1 second after the stimuli. The EMG signals were also monitored continuously on an oscilloscope (Tectronix, Inc., Wilsonville, OR) and on computers so that any activity in the test muscles prior to the stimuli was obvious. Participants were always instructed to relax the test muscles before stimulation because our aim was to deliver stimuli to muscles at rest so that we could compare responses in SCI and control participants under the same conditions.

### Cutaneous nerve stimulation

Interlimb reflexes were evoked by electrical stimulation of two nerves that are largely cutaneous at the sites of stimulation: the superficial peroneal nerve on the dorsum of the foot and the radial nerve at the wrist. Stimulating electrodes were placed ~4 cm apart on the dorsum of the right foot of each participant to stimulate the superficial peroneal nerve (SPN). The left foot was used in one participant because no EMG was evoked with stimulation of the right foot using 80 mA which is close to the mean intensity used for SCI participants. The radial nerve (RN) was stimulated via electrodes placed ~4 cm apart along the radial side of the right wrist. Stimuli were delivered to the electrodes from a constant current stimulator (Digitimer DS7H, Digitimer Ltd., Hertfordshire, England) controlled by Fystat software (Umeå University, Sweden).

Reflexes were evoked by delivering trains of stimuli (range: 23–30 trains, 5 pulses at 300 Hz, pulse duration: 200 μs; [25]) to the SPN and then to the RN in a second set of trials. Participants were encouraged to relax prior to and during each trial. Trains of stimuli were delivered approximately 30 s apart or after any evoked EMG had subsided.

Stimulus intensity was set as multiples of the threshold (radiation threshold; RT) at which the participant felt parathesiae radiate away from the stimulus site [25]. The intensity of stimulation was increased so as to evoke a strong cutaneous sensation but was not felt as noxious. Nine of the 17 participants with SCI perceived the stimuli (53%). For SPN stimulation, the mean (± SEM) intensity used to evoke reflexes across participants with SCI who perceived the stimulation was 74 ± 3 mA (3.1 ± 0.4 x RT) and for those participants with SCI who did not perceive the stimulation, the mean intensity was 76 ± 2 mA, whereas a mean of 65 ± 8 mA (3.3 ± 0.7 x RT) was used for able-bodied control participants. For RN stimulation the corresponding stimulus intensities were 54 ± 8 mA (3.9 ± 0.5 x RT) and 73 ± 7 mA for participants with SCI who did and did not perceive the stimuli, respectively, and 62 ± 10 mA (4.5 ± 0.3 x RT) for able-bodied control participants. For both SPN and RN stimulation, no significant differences were found between the 3 groups with respect to the mean absolute stimulus intensity used or between the mean radiation thresholds for control participants and the participants with SCI who perceived the stimuli (ANOVA; p>0.05).

### Definition of interlimb reflexes

The side of the body where the stimuli were delivered was termed ipsilateral, the opposite, contralateral. If the average rectified EMG increased above the pre-stimulus resting baseline for 10 ms or longer, it was considered a reflex response. A within-limb reflex occurred when EMG was evoked in any one of the four test muscles in the stimulated limb. An interlimb reflex occurred when EMG was evoked in any one of the other twelve test muscles in the three non-stimulated limbs (e.g. in an ipsilateral or contralateral arm or contralateral leg muscle in response to ipsilateral SPN stimulation).

**Fig 1:**
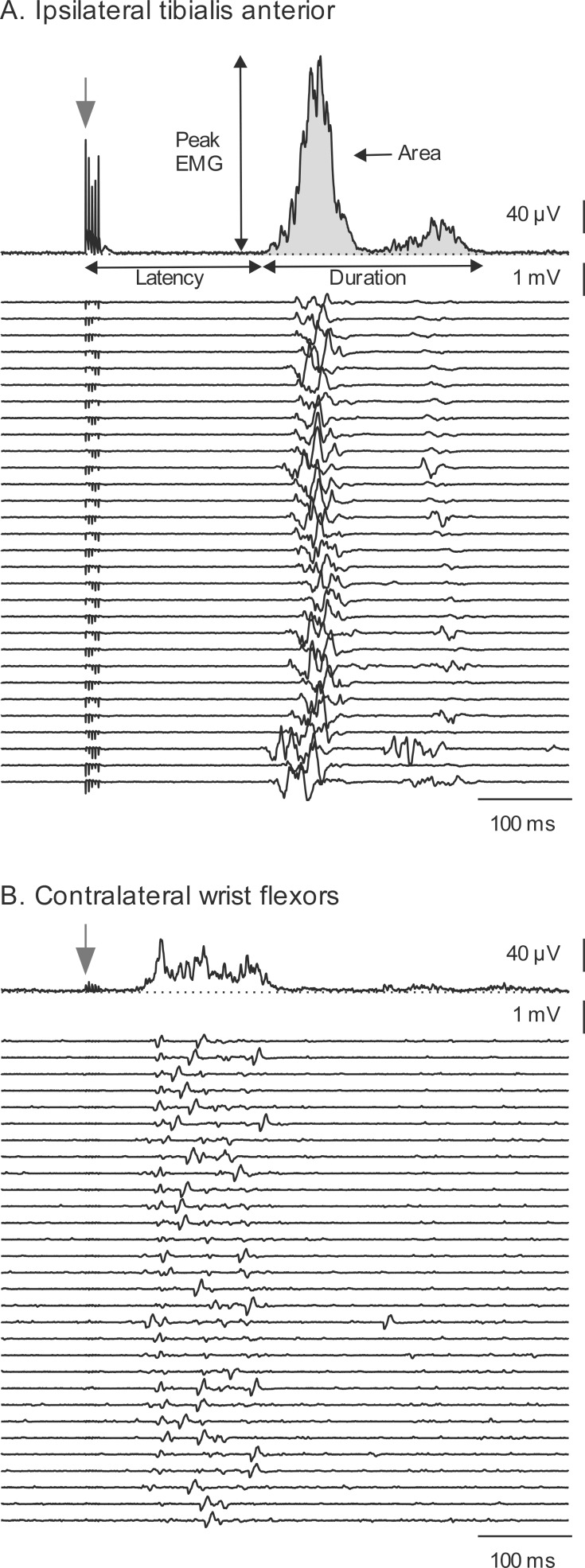
A within-limb and an interlimb reflex evoked in a participant with SCI. EMG was evoked in (A) the ipsilateral tibialis anterior and (B) contralateral wrist flexors with superficial peroneal nerve stimulation (within-limb and interlimb reflex respectively). The top trace in each panel depicts the rectified averaged EMG from the 30 trials of EMG rastered below. Latency, duration, peak and integrated EMG (shaded) were measured for each reflex as shown in A. Onset of stimulus train is indicated by the stimulus artefact and an arrow.

Three sub-types of interlimb reflex responses were also defined. Those reflexes that appeared at a higher segmental level than the spinal level of primary sensory input were termed ascending interlimb reflexes (e.g. activation of arm muscles by SPN stimulation). Those reflexes that appeared in muscles innervated by motoneurons at a lower segmental level than the spinal level of primary sensory input were termed descending interlimb reflexes (e.g. activation of leg muscles in response to RN stimulation). Reflexes evoked in the opposite limb to that stimulated were termed contralateral interlimb reflexes. Fig 1 shows examples of a within-limb reflex (Fig 1A) and an ascending interlimb reflex (Fig 1B) evoked by SPN stimulation in a participant with SCI.

### Data Analysis

Analysis of surface EMG signals was performed offline using Zoom software (Umeå University, Sweden). All trials for each of the 16 channels of EMG were rectified and averaged. For each reflex response, measurements were made of latency (time between the first stimulus and the beginning of the EMG), duration (time between the beginning and end of the reflex EMG), and area (integrated EMG for the duration of the reflex; see Fig 1A). The integrated EMG was then normalized to the duration of the reflex to provide the mean reflex EMG magnitude during the response.

Reflex incidence in response to SPN and RN stimulation was determined for each of the 16 muscles recorded. The reflex incidence was then grouped into an overall measure of incidence for within-limb reflexes (ipsilateral leg for SPN stimulation and ipsilateral arm for RN stimulation), and interlimb reflexes which included, the contralateral limb reflex and ascending and descending reflexes in the ipsi- and contralateral arms or legs.

### Statistics

Chi-square tests were performed to test where the incidence of within-limb and interlimb reflexes differed between SCI and control groups, between limbs and between stimulation sites. Multiple logistic regressions were performed to assess whether ascending and descending interlimb reflex incidence could be predicted by group (SCI or able-bodied control), stimulation site (SPN or RN), or muscle. Data are presented as median (± interquartile range) for latency, duration and EMG magnitude. Reflex latency, duration, and mean EMG magnitude were compared for SCI and control groups using a 2-way analysis of variance (ANOVA) on ranks, as data were not distributed normally, with muscle and group as factors and Tukey post-hoc tests. Multiple linear regressions were performed to assess whether reflex latency, duration and magnitude could be predicted by group, stimulation site, or muscle. Statistical significance was set at p<0.05.

## Results

Ascending, descending and contralateral interlimb reflex responses were evoked in muscles of participants with SCI and able-bodied control participants with both superficial peroneal nerve (SPN) and radial nerve (RN) stimulation.

**Fig 2:**
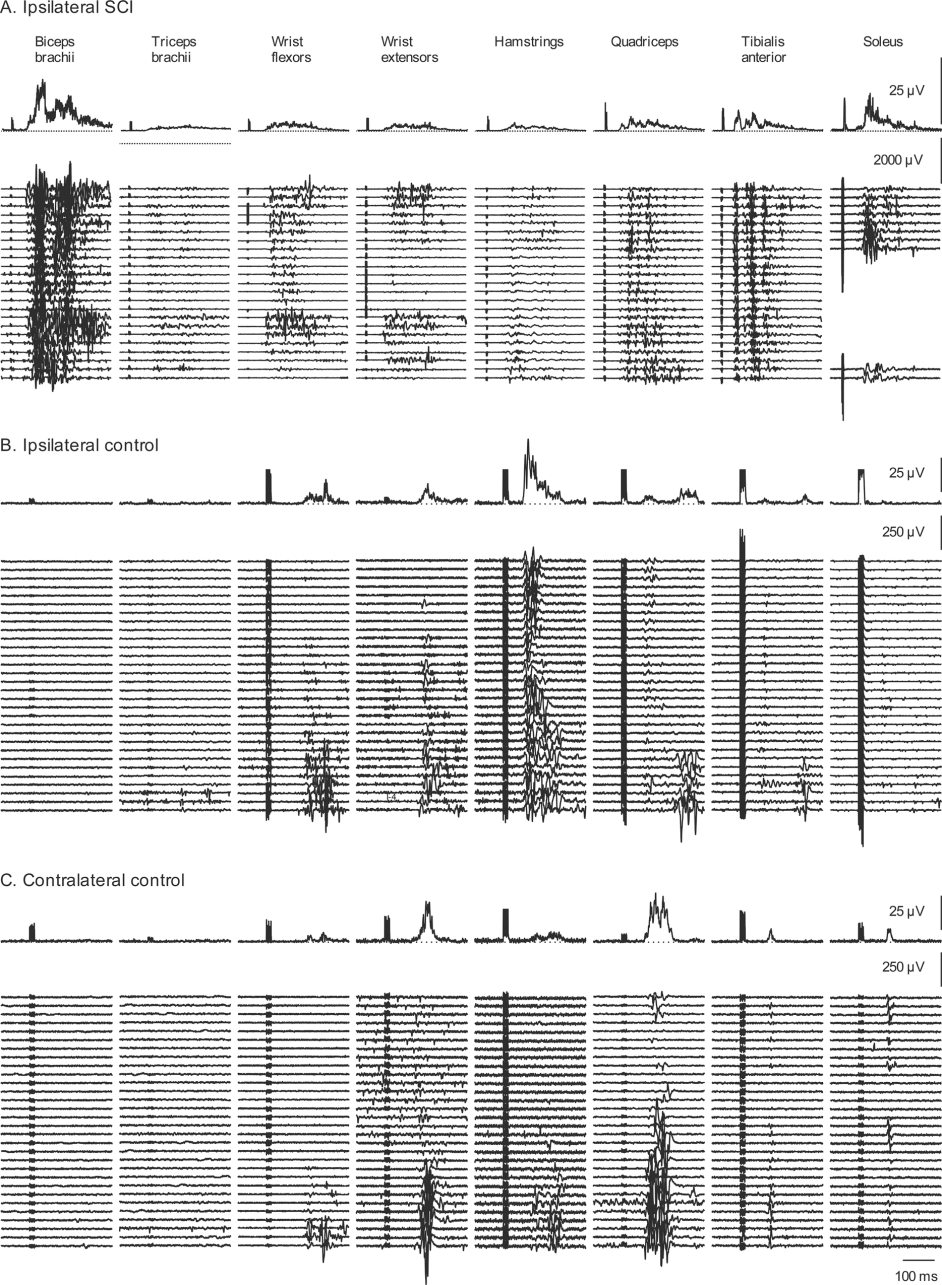
Interlimb and within-limb reflexes with SPN stimulation. EMG evoked by SPN stimulation in 8 ipsilateral muscles of a participant with SCI (A) and each of the 16 muscles (ipsilateral and contralateral) of one control participant (B,C). There were no responses present in contralateral muscles of the participant with SCI. Therefore, they are not shown. The top trace in each panel depicts the rectified averaged EMG from all of the trials of stimulation (n = 23) rastered below. In A, trials that included artefacts from cable movement were removed from the soleus records. Stimuli occurred at the onset of the stimulus artefact visible in each average.

Fig 2 shows raster plots of the EMG recorded from 8 ipsilateral muscles in response to SPN stimulation for one participant with SCI (Fig 2A) and 16 muscles of one control participant (Fig 2B and 2C) together with the rectified averaged EMG for each muscle above the single trials. For the participant with SCI, ascending interlimb reflexes were present in all four ipsilateral arm muscles, including biceps brachii and triceps brachii. All four leg muscles showed within-limb reflexes. There were no responses in contralateral arm or leg muscles in this participant (not shown). In contrast, for the able-bodied participant, ascending interlimb EMG responses occurred in ipsilateral and contralateral wrist flexors and extensors, and contralateral interlimb responses were observed in every contralateral leg muscle studied (hamstrings, quadriceps, tibialis anterior and soleus). Within-limb reflex responses were also evident in all four muscles tested.

**Fig 3:**
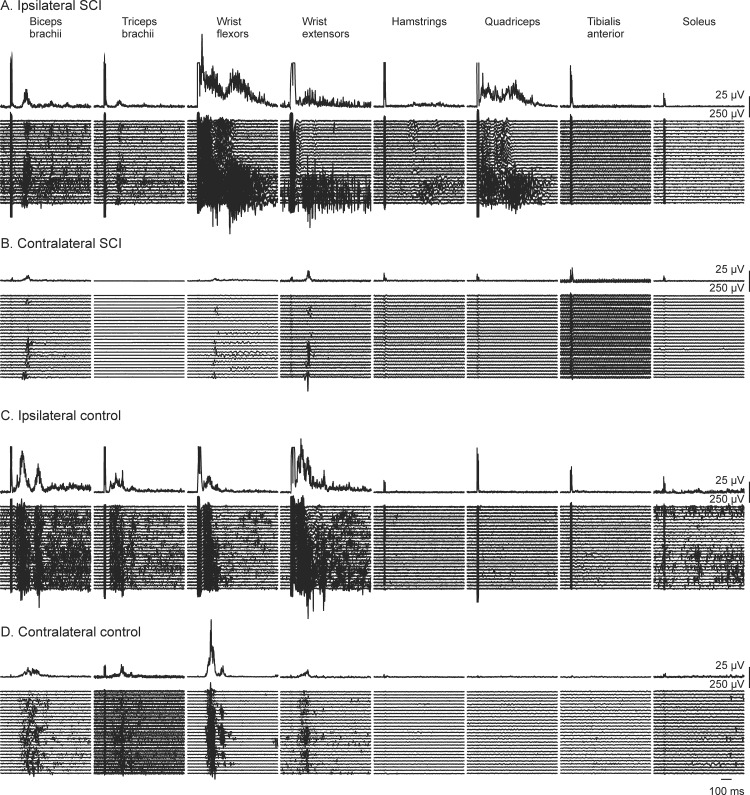
Interlimb and within-limb reflexes with RN stimulation. EMG evoked by radial nerve stimulation in 16 muscles (8 muscles recorded ipsilaterally, 8 contralaterally) of a participant with SCI (A,B) and one control participant (C,D). The top trace in each panel depicts the rectified averaged EMG from all of the trials of stimulation (n = 30) rastered below. Stimuli occurred at the onset of the stimulus artefact visible in each average.

Fig 3 shows raster plots of the EMG recorded from the 16 muscles in response to RN stimulation of one participant with SCI and one control participant together with the average of the rectified EMG for each muscle above the single trials. Contralateral interlimb reflexes occurred in contralateral arm muscles in both participants shown here, but descending interlimb reflexes were observed only in the ipsilateral leg muscles of the participant with SCI.

The trains of stimuli to the cutaneous nerves sometimes resulted in sustained activation (e.g. wrist flexors and extensors, Figs 2A and 3A), multiple bursts of EMG (e.g. biceps brachii, Figs 2A and 3C; tibialis anterior, Fig 2A) or activation of single motor unit potentials (e.g. wrist flexors, Fig 1B; wrist extensors, Fig 3D).

### Incidence of interlimb and within-limb reflexes

**Fig 4:**
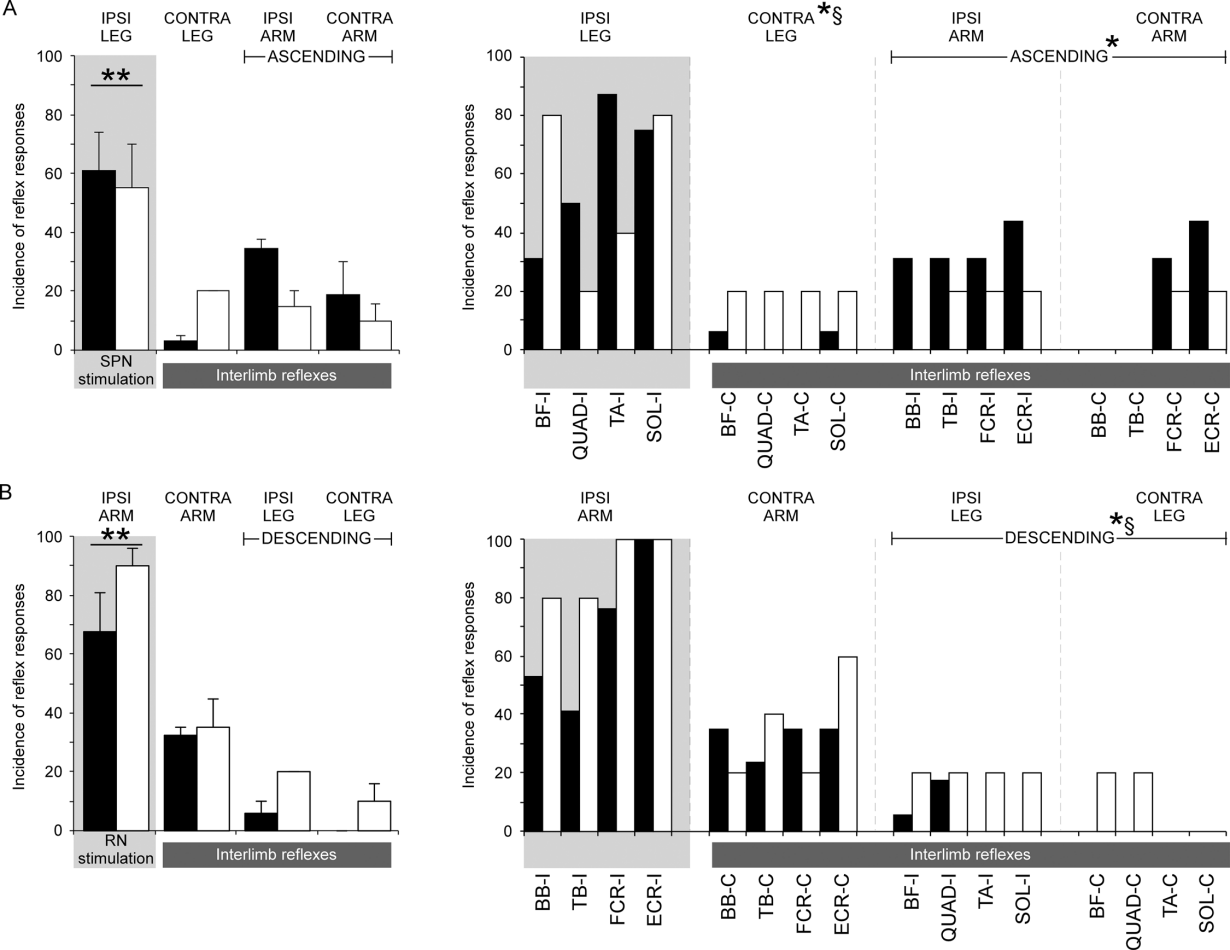
Incidence of within-limb, interlimb, and muscle reflexes. Incidence of limbs (left) and muscles (right) in which reflexes were evoked in participants with spinal cord injury (SCI) (filled bars; n = 17) and able-bodied (AB) control participants (open bars; n = 5) by SPN stimulation (A) and RN stimulation (B). Within-limb reflex data are shaded. Muscles from the arm are indicated by BB–biceps brachii, TB–triceps brachii, FCR–flexor carpi radialis, and ECR–extensor carpi radialis, and muscles from the leg are indicated by BF–biceps femoris, QUAD–quadriceps, TA–tibialis anterior, and SOL–soleus. ** indicates significant differences in incidence between within-limb and interlimb reflexes § indicates significant differences in incidence between SCI and AB control groups. * indicates a significant relationship between having a SCI and the outcome (assessed by logistic regression).

Fig 4A shows the incidence of reflex responses to SPN stimulation for SCI (filled bars) and control participants (open bars) by limb (left) and muscle (right). Stimulation of the SPN evoked a response in at least one muscle in 16 of the 17 participants with SCI and in all 5 control participants. Overall, in response to SPN there was no difference between SCI and control groups in the incidence of within-limb (61% and 55%, respectively, p = 0.83) or interlimb reflexes (19% and 15%, respectively, when the three unstimulated limbs were grouped together, p = 0.58), although the incidence of within-limb reflexes was higher than interlimb reflexes across both groups (p<0.0001, Table 2). The incidence of ascending interlimb reflexes in the arm muscles evoked by SPN stimulation in participants with SCI (34% and 20% for ipsilateral and contralateral arms, respectively) was about double that in control participants (15% and 10% for ipsilateral and contralateral arms, respectively) but this did not reach significance with the chi-squared test (p = 0.087). However, a logistic regression showed that having a SCI was positively related to the incidence of ascending interlimb reflexes with an odds ratio of 3.3 (1.1–10.3, 5%-95%CI, p = 0.04). The contralateral interlimb reflex to leg muscles was lower after SCI (3%) than for the control group (20%, p = 0.03) for SPN stimulation and this finding was supported by the logistical regression with an odds ratio of 0.12 (0.02–0.72, 5%-95%CI, p = 0.02).

**Table 2:** Incidence of within-limb and interlimb reflexes.

	SCI (n = 17)		AB (n = 5)	
	SPN	RN	SPN	RN
Within-limb	61%^†^ (n = 15)	68%^†^* (n = 17)	55%^†^ (n = 5)	90%^†^ (n = 5)
Interlimb	19% (n = 12)	13% (n = 17)	15% (n = 2)	22% (n = 5)
Ascending Interlimb	34% (I)* 20% (C)*		15% (I) 10%(C)	
Descending Interlimb		6% (I)* 0% (C)*		20% (I) 10% (C)
Contralateral Interlimb	3%*	32%	20%	35%

Table 2: Incidence of within-limb, interlimb reflexes (ascending, descending, and contralateral grouped). Interlimb reflexes were further categorised as ascending, descending and contralateral in participants with spinal cord injury (SCI) and able-bodied control participants (AB). Ascending and descending reflexes are divided into ipsilateral (I) and contralateral (C) reflexes. The numbers of participants, from a total of 17 for SCI and 5 for AB that demonstrated within-limb and interlimb reflexes are shown in brackets.

^†^ indicates a difference from the overall interlimb reflex incidence

* indicates a significant relationship between having a SCI and the outcome (assessed by logistic regression)

Responses to RN stimulation were observed in at least one muscle in all 17 participants with SCI and all 5 control participants (Fig 4B). For RN stimulation, within-limb reflexes occurred with high incidence in both participants with SCI (68%) and control participants (90%). The within-limb incidences were not significantly different between groups (p = 0.09, Table 2, Fig 4B, left panel). For both groups, the incidence of within-limb reflexes was higher than the incidence of interlimb reflexes (p<0.001), although the incidence of interlimb reflexes was not significantly different between SCI and control groups (13% and 22%, respectively when the three unstimulated limbs were grouped together, p = 0.13). In contrast to the higher incidence of ascending interlimb reflexes evoked by SPN stimulation in participants with SCI, we found the descending interlimb reflex responses in leg muscles that were evoked by RN stimulation occurred significantly less in the SCI group (6% and 0% for ipsilateral and contralateral legs, respectively) than in the control group (20% and 10% for ipsilateral and contralateral legs, respectively, p = 0.012). This was confirmed by the multiple logistic regression that showed that having a SCI was negatively related to the incidence of descending interlimb reflexes with an odds ratio of 0.2 (0.06–0.7, 5%-95%CI, coefficient = -1.6, p = 0.009). Contralateral arm responses occurred with similar incidences in the SCI and control groups (mean 32% and 35%, respectively, p = 0.96).

The overall incidence of interlimb reflexes evoked by RN stimulation (18%) was similar to that evoked by SPN stimulation (15%, p = 0.34) for both the SCI group (13% vs 19% for RN vs SPN stimulation, respectively, p = 0.10) and the control group (22% vs 15% for RN vs SPN stimulation, respectively, p = 0.48). The overall incidence of ascending interlimb reflexes evoked by SPN (mean 20%) was larger than the incidence of descending interlimb reflexes evoked by RN stimulation (mean 9%, p<0.001). This was largely due to the increased incidence of ascending reflexes in participants with SCI.

### Reflex Latency, Duration and Magnitude

**Fig 5:**
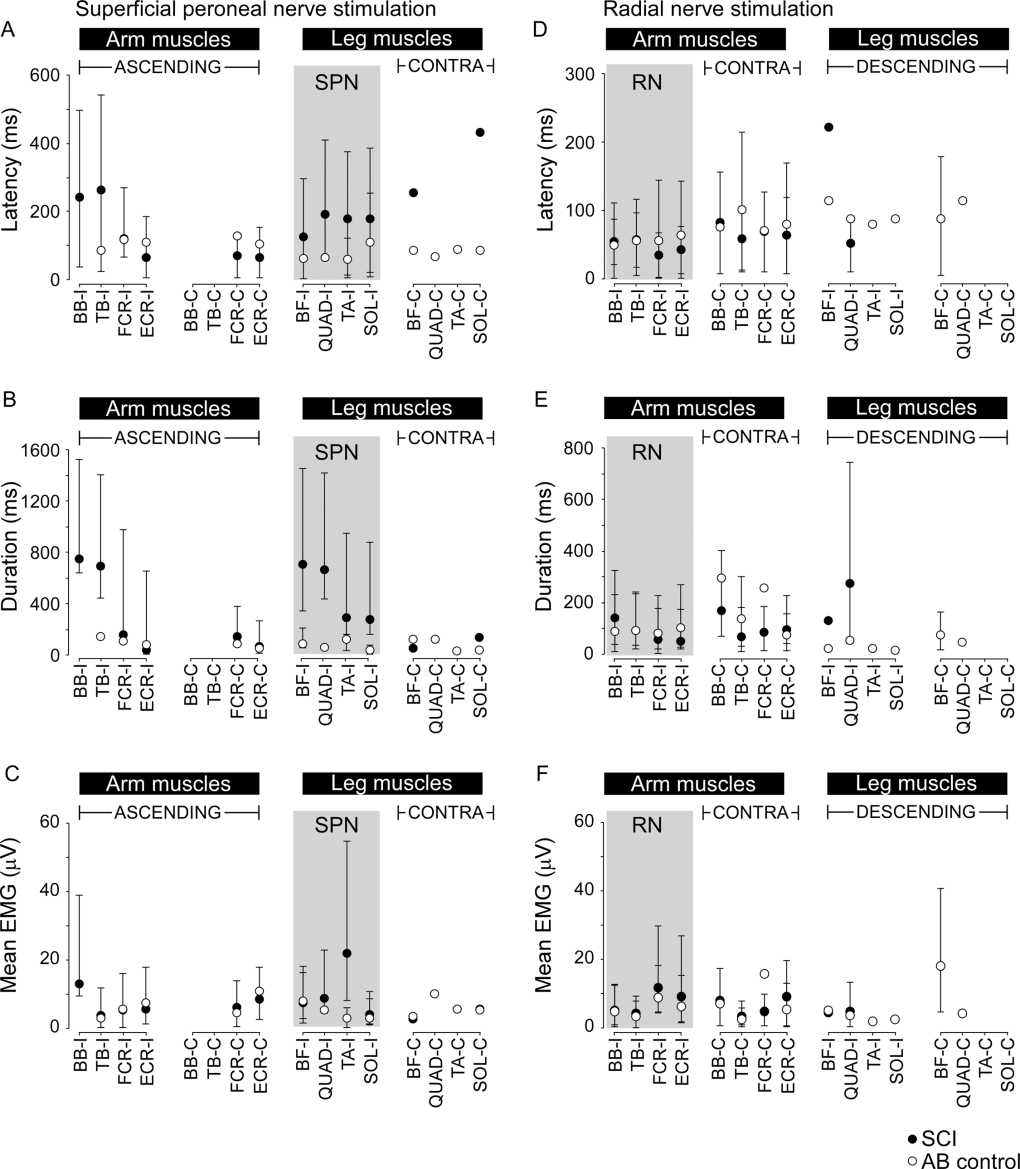
Reflex latency, duration and mean EMG amplitude for all muscles. Data (median ±IQ range) for reflex latency (A and D), duration (B and E) and mean amplitude (C and F) are shown for SCI (filled symbols) and control groups (open symbols). Data for each muscle in both the arm and leg on the ipsilateral (I) and contralateral (C) sides are shown in each panel for SPN (A-C) and RN (D-F) stimulation. The grey shaded box indicates the muscles with within-limb reflexes for SPN (left panels) and RN (right panels) stimulation. Arm muscles are indicated by BB–biceps brachii, TB–triceps brachii, FCR–flexor carpi radialis, and ECR–extensor carpi radialis, and leg muscles are indicated by BF–biceps femoris, QUAD–quadriceps, TA–tibialis anterior, and SOL–soleus. Interlimb reflexes termed ascending, descending and contralateral (contra) are also indicated.

Fig 5 shows the median values (± interquartile range) for reflex latency, duration and mean EMG magnitude for the different muscles studied in response to SPN and RN stimulation in SCI and control groups.

Two-way ANOVAs with muscle and group as factors showed that across all reflex responses to SPN stimulation the median onset latency was two times longer for the SCI group (169 ms, filled circles, Fig 5A) than the control group (85 ms, open circles, Fig 5A, p<0.001). Of note though, short-latency ascending interlimb reflexes (~65 ms) were observed in contralateral wrist extensor and flexor muscles evoked by SPN stimulation in 7 participants with SCI. The median duration of the EMG response was four times longer for the SCI group (245 ms, filled circles, Fig 5B) than the control group (median duration 57 ms, filled circles, Fig 5B, p<0.001). While EMG area was significantly larger for the SCI group (p<0.001) due in part to the increased duration, the overall magnitude of the reflexes (mean EMG) was 1.5 times larger for the SCI group but did not differ significantly between groups (p = 0.054, Fig 5C). Multiple linear regression analysis showed that a longer latency reflex and reflex duration, but not size, could be predicted by having a SCI (p<0.001).

For RN stimulation, the median reflex latency (57 ms and 75 ms for SCI and control groups, respectively, p = 0.85, Fig 5D), median duration (91 ms and 79 ms, for SCI and control groups, respectively, p = 0.27, Fig 5E) and the median mean EMG magnitude of the reflexes (1.5 times larger overall for the SCI group, p = 0.08, Fig 5F), were not significantly different between SCI and control groups and could not be predicted by SCI through multiple linear regression analysis.

The mean EMG data for the reflex responses are absolute values compared across participants and muscles. To account for atrophy of the paralyzed muscles in participants with SCI, we normalized the magnitude of the reflex EMG to the maximal M wave for tibialis anterior in a subset of 4 participants with SCI and one able-bodied control participant. In this subset, the absolute size of the reflex was 5 times larger in tibialis anterior for the SCI group but when normalized to the maximal M wave the reflex response was 20 times larger than the control participant. This shows in principle that the size of the reflex responses may be underestimated four-fold for the SCI group as only absolute magnitude is compared for all of our participants. However, the extent of the underestimation will depend on the degree of muscle atrophy and we cannot extrapolate this finding due to the small size of the subset.

## Discussion

Excitatory interlimb reflexes were evoked with similar incidence in participants with chronic SCI and in neurologically intact participants with stimulation of the superficial peroneal nerve on the dorsum of the foot and with stimulation of the radial nerve at the wrist. However, ascending interlimb reflexes tended to occur with a higher incidence in participants with SCI, while descending interlimb reflexes occurred with a higher incidence in able-bodied participants. With SPN stimulation, interlimb reflexes had longer latencies and longer durations in the SCI group but with RN stimulation, latency and duration were similar for both groups. The potential mechanisms underlying the differences seen in interlimb reflexes after SCI will be discussed.

The majority of previous studies of interlimb reflexes in able-bodied individuals have demonstrated inhibitory interlimb reflexes [25,30,31] where it is necessary to test during voluntary contraction of muscles or reflex excitation because inhibitory reflexes cannot be assessed when muscles are tested at rest even though they may occur. A smaller number of studies in able-bodied individuals have shown only facilitatory effects of interlimb reflexes [23], or both excitatory and inhibitory interlimb reflexes that depend on limb position [35] or direction of joint rotation [32]. The differences in results across studies suggest reflex effects are state dependent [36].

Like Calancie et al. [2–5], we have demonstrated short-latency excitatory ascending interlimb reflexes at rest in participants with SCI, with higher incidence than for able-bodied participants. The proposed mechanism for the occurrence of interlimb reflexes after injury is that they emerge as a result of new functional connections which are made by ascending afferents from lower limb muscles that sprout and connect with upper limb neurons that have lost their descending inputs (due to the more rostral spinal cord injury [4]). New intraspinal connections have been demonstrated in animal studies [8–13], (for review see [14]). In addition to ascending interlimb reflexes, we have also demonstrated excitatory descending interlimb reflexes in four participants with motor complete SCI, although at lower incidence than in able-bodied participants. All of these responses were evoked in proximal leg muscles although descending voluntary responses were not detectable when tested by manual muscle analysis for the AIS assessment. Both excitatory ascending and descending interlimb reflexes were present in able-bodied control participants at rest. Thus, our data also support the view that existing interlimb reflex connections are retained after SCI and in some cases may be further enhanced by new intraspinal connections, particularly for ascending interlimb reflexes.

Previous studies have not been able to demonstrate interlimb reflexes in able-bodied participants with their muscles at rest [3,4]. This may be due to differences in the nerve afferent classes that have been stimulated. We used relatively high intensity stimulus trains (5 pulses at 300 Hz) of cutaneous nerves (SPN and RN) based on methods shown previously to initiate inhibitory interlimb reflexes in able-bodied participants during voluntary contractions of the target muscles [25] and this stimulation is likely to activate both small and large diameter cutaneous afferents. The experiments by Calancie et al. also used relatively high intensities of stimulation, but they stimulated the tibial nerve (3–4 pulses at 500 Hz [4]), a mixed motor and sensory nerve. This stimulation will cause both a muscle twitch and activation of muscle afferents, which did not occur with SPN and RN stimulation in our study.

### Interlimb reflex latencies

The electrical stimulus intensities used in the current study are likely to have activated both large diameter (fast conducting) and small diameter (slower conducting) cutaneous afferent fibers. Short-latency reflexes (defined as those that began at <100 ms) occurred for both SCI and control groups, particularly in contralateral arm muscles in response to RN stimulation. The short-latency reflexes shown here and in previous studies in individuals with SCI [2–6] and able-bodied individuals [23–25,27,29,33] suggest that fast-conducting large-diameter cutaneous afferents may be responsible for these reflexes and that fast-conducting intraspinal pathways are involved (e.g. the spinocervical or propriospinal tracts). Some short-latency interlimb reflexes in the current study appear in both SCI and control participants at 30–40 ms even when the within-limb reflex occurred at 170–180 ms (Fig 1). The spinocervical tract receives inputs from large numbers of cutaneous afferents and mainly has strong ipsilateral projections [37,38]. Long propriospinal tracts make ipsilateral and contralateral connections between forelimbs and hindlimbs and have been demonstrated in the cat [39–41] and in human upper limbs [42–45] and are likely to have a role in neural recovery after spinal cord injury (for review see [46]). It is possible that the short-latency interlimb reflexes shown here are evidence for ascending and descending fast conducting propriospinal pathways between the arms and legs in human participants.

Longer latency reflexes (> 100 ms), observed in both groups, are consistent with the flexor-withdrawal reflex which is mediated via activation of high threshold slowly conducting afferents (e.g. C-fibers or Aδ-fibers, [47–49]). The flexor-withdrawal reflex appears at longer latencies in individuals with complete SCI than in controls [50,51], (see also [52]). After SCI, conduction slowing may occur on either the afferent or efferent limb of the reflex due to changes in myelination in central or peripheral nervous system axons [53,54]. Another possibility is that the long latency reflexes involve multiple synapses in the spinal cord. Some of the variability we see in the latencies across and within individuals with SCI may be because there are many different possible combinations of changes in afferent, intraspinal and efferent fibers that could be involved in the reflex.

All of our participants with SCI were assessed as motor complete (AIS A or B). However, for able-bodied participants, there is a possibility that the long-latency for within limb and/or interlimb responses contain a supraspinal input, despite the participants sitting at rest. Voluntary reactions in the lower limb muscles can occur in response to electrical stimulation of the foot at latencies greater than 112 ms (mean 136 ms, [55]) although there may be cortical contributions to lower limb reflexes as early as ~80 ms [56]. However, our instruction to remain relaxed should minimize these potential effects. Startle responses in neck muscles to somatosensory stimuli at the wrist mediated by bulbospinal pathways occur at >80 ms [57], but these ought to habituate and should not persist across multiple successive trials. Despite the possibility of a contribution from descending pathways in the able-bodied participants, interlimb reflex latencies were only consistent across trials in any given muscle, but they were inconsistent across limbs within an individual (e.g. a response in the leg could occur earlier than one in the arm). These types of responses are unlikely to be produced voluntarily or through other descending (supraspinal) pathways. Although we cannot be certain of the spinal nature of long-latency interlimb reflexes in the able-bodied control group, we know that functional spinal reflex connections between upper and lower extremities exist in able-bodied individuals ([25,26,35,58], for review see [59]).

### Interlimb reflex duration and size

For SPN stimulation, the interlimb reflexes were of longer duration for participants with SCI. This was not the case for RN stimulation suggesting that ascending interlimb reflexes are more prolonged after SCI due to increased regional spinal excitability. While interlimb reflexes were 1.5 times larger in absolute magnitude for both SPN and RN stimulation for SCI versus control groups, this was not a significant increase. However, there is a high likelihood that if the EMG was normalized to account for muscle atrophy, a common characteristic of muscles paralyzed by SCI [60,61], the relative magnitude of the interlimb reflexes would be increased in participants with SCI. In our study, normalization of the mean EMG to the maximal evoked M wave to adjust for reductions in maximal M waves was limited to only a subset of subjects.

Potential mechanisms for the increases in the magnitude and duration of reflexes after SCI include reductions in inhibition or increases in excitation caused by removal of descending input [7,21,62,63], changes in synaptic strength [5,60], and/or sprouting of existing axons to form new connections [2,64]. Another possible contributor to increased ascending interlimb reflexes may be the re-emergence of persistent inward currents in motoneurons below the level of the injury that make the motoneurones hyperexcitable [for review see 7]. The recovery of persistent inward currents after SCI is thought to be due to an increase in “self-activation” of monoaminergic receptors [65]. However, it would be expected that this mechanism should apply to both ascending and descending interlimb reflexes and we only see an increase in the ascending interlimb reflexes after SCI.

### Conclusion

While our data do not allow us to comment on the exact neural pathways underlying the interlimb and within-limb responses to sensory nerve stimulation, overall, our findings show that functional interlimb connections exist in able-bodied control participants and participants with SCI. However, for participants with SCI, the combined findings for ascending interlimb reflexes (higher in incidence, longer in duration, and likely increased in magnitude), are consistent with an alteration in the balance of inhibition and excitation across existing interlimb connections within the spinal cord (for review see [19]). This may be due to the strengthening or ‘unmasking’ of existing inputs, or to new connections from axonal sprouting [66,67], which may affect the reflex responses of each participant with SCI differently. New growth is thought to be highly localized [68,69] and is observed in experimental animal models after 3 weeks [12], and for humans has been implicated to explain the emergence of interlimb reflexes at 5.5 months after injury [4], and their further increase in incidence with time after injury [5]. However, new growth as described above does not explain the demonstration of descending interlimb reflexes in participants with SCI or the occurrence of interlimb reflexes in able-bodied participants.

## Supporting Information

S1 Text Individual data from all participants. All measures of averaged reflexes are shown for each muscle and each participant.
(XLSX)

## Abbreviations

BB: biceps brachii
BF: biceps femoris
C: contralateral
EMG: electromyograph
ECR: extensor carpi radialis
FCR: flexor carpi radialis
I: ipsilateral
QUAD: quadriceps
RN: radial nerve
RT: radiation threshold
SOL: soleus
SCI: spinal cord injury
SPN: superficial peroneal nerve
TA: tibialis anterior
